# Myocardial Bridging Increases the Risk of Adverse Cardiovascular Events in Patients without Coronary Atherosclerosis

**DOI:** 10.3390/life14070811

**Published:** 2024-06-26

**Authors:** Tsung-Lin Yang, Wen-Rui Hao, Chun-Chao Chen, Yu-Ann Fang, Hsin-Bang Leu, Ju-Chi Liu, Shing-Jong Lin, Jiun-Lin Horng, Chun-Ming Shih

**Affiliations:** 1 Graduate Institute of Clinical Medicine, College of Medicine, Taipei Medical University, Taipei 110, Taiwan; 151017@h.tmu.edu.tw; 2 Department of Internal Medicine, School of Medicine, College of Medicine, Taipei Medical University, Taipei 110, Taiwan; b8501043@tmu.edu.tw (W.-R.H.); b101092035@tmu.edu.tw (C.-C.C.); liumdcv@s.tmu.edu.tw (J.-C.L.); 3 Division of Cardiology, Department of Internal Medicine, Taipei Medical University Hospital, Taipei 110, Taiwan; sjlin@vghtpe.gov.tw; 4 Cardiovascular Research Center, Taipei Medical University Hospital, Taipei 110, Taiwan; 5 Taipei Heart Institute, Taipei Medical University, Taipei 110, Taiwan; 6 Division of Cardiology, Department of Internal Medicine, Shuang Ho Hospital, Taipei Medical University, New Taipei City 235, Taiwan; 7 School of Medicine, National Yang Ming Chiao Tung University, No. 155, Section 2, Linong Street, Taipei 112, Taiwan; hbleu@vghtpe.gov.tw; 8 Division of Healthcare and Management, Healthcare Center, Taipei Veterans General Hospital, Taipei 112, Taiwan; 9 Department of Anatomy and Cell Biology, School of Medicine, College of Medicine, Taipei Medical University, Taipei 110, Taiwan; jlhorng@tmu.edu.tw

**Keywords:** myocardial bridging, cardiovascular event, long-term effects, nationwide study

## Abstract

**Background:** Myocardial bridging (MB) is a congenital coronary anomaly and an important cause of chest pain. The long-term effects of MB on cardiovascular events remain elusive. **Methods:** We used the National Health Insurance Research Database of Taiwan to conduct an analysis. All patients who had undergone coronary angiography were considered for inclusion. The primary endpoint was a composite of nonfatal myocardial infarction, nonfatal ischemic stroke, and cardiovascular death. **Results:** We identified 10,749 patients from 2008 to 2018 and matched them with an equal number of controls by propensity-score matching. The mean follow-up period was 5.78 years. In patients without coronary artery disease, MB increased the risk of the composite endpoint (hazard ratio [HR]: 1.57, 95% confidence interval [CI]: 1.44–1.72, *p* < 0.001), which was driven by increased risks of nonfatal myocardial infarction and cardiovascular death. In patients with significant coronary artery disease, MB did not increase the risk of major adverse cardiovascular events. MB was identical to insignificant coronary artery disease from the viewpoint of clinical outcomes. **Conclusions:** The presence of MB significantly increases cardiovascular risks in patients with normal coronary vessels. Atherosclerotic coronary artery disease mitigates the effect of MB on cardiovascular outcomes. MB can be considered an insignificant coronary artery disease equivalent.

## 1. Introduction

Coronary arteries are blood vessels originating from the aorta that go beyond the surface of myocardium in the epicardial space. Myocardial bridging (MB) is a common coronary anomaly in which these arteries embed within the muscular layers of the heart. This condition leads to vascular compression during systole, which partially compromises coronary flow during the diastolic phase [1]. MB’s prevalence, which has been estimated to be 33% to 42% and 20% to 25% from autopsy and intravascular ultrasound (IVUS) data, respectively [2,3,4,5], tends to be higher in men [2]. No randomized trial has been conducted to establish evidence-based recommendations for MB management. Beta adrenergic antagonists or non-dihydropyridine calcium channel blockers, due to negative chronotropic and inotropic effects, are recommended for symptomatic relief for MB. Percutaneous coronary intervention, stenting, bypass grafting, or myotomy are generally considered for patients who are unresponsive to optimal medication [1,6,7]. MB segments predominantly affect the left anterior descending coronary arteries [8] and are prone not to undergo atherosclerosis because of certain mechanisms that ameliorate inflammation [9,10,11]. Nevertheless, atherosclerotic changes often develop proximal to the MB region due to blood flow stasis [5]. The clinical manifestation of MB varies; MB can be asymptomatic or associated with severe symptoms such as acute coronary syndrome, fatal arrhythmia and sudden cardiac death [12,13]. MB is typically associated with exertional chest pain, which is difficult to differentiate from symptoms of atherosclerotic coronary artery disease. Many researchers have attempted to identify and characterize MB by using noninvasive tools; however, only coronary computed tomographic angiography has achieved adequate diagnostic results [14]. Although it is generally considered benign, MB has been linked to serious outcomes such as cardiac arrest and fatal arrhythmia in certain populations [15,16], including patients with hypertrophic cardiomyopathy [17] or Takotsubo cardiomyopathy [18], and patients who have undergone cardioverter defibrillator implantation [12]. MB is also associated with an increased risk of atherosclerosis and reduced long-term survival after heart transplantation [19]. These observations underscore MB’s potential role in major adverse cardiac events (MACEs). Large-scale study with long-term follow-up investigating the effects of MB on MACEs is lacking. The current study aimed to compare the clinical trajectories of patients with and without MB, particularly with a focus on MACEs over long-term follow-up.

## 2. Materials and Methods

### 2.1. Database

The National Health Insurance program of Taiwan was established in 1996 and covers >99% of the Taiwanese population. Subsequently, the Ministry of Health and Welfare established the National Health Insurance Research Database (NHIRD) for the purposes of scientific research and maintaining public health. This database contains the deidentified data of >23 million individuals in Taiwan, including information regarding various clinical parameters, such as sex, age, details of outpatient visits, hospitalization history, diagnoses according to International Classification of Diseases (ICD) codes, examinations, prescribed medications, surgical or other medical procedures, and survival status.

### 2.2. Study Population

We established a MB group, which included all patients who had undergone coronary angiography between 1 January 2008 and 31 December 2018 and were subsequently given a diagnosis of MB. Patients who had undergone coronary angiography but did not receive a diagnosis of MB formed a non-MB group. We matched these groups at a 1:1 ratio by using propensity scores, with consideration of factors such as age, sex, underlying medical conditions, and medication history.

Propensity-score matching was performed to reduce the effects of confounding variables and to improve the accuracy of our estimates regarding the effects of specific treatments or variables (MB in the current study). We compiled data regarding clinical covariates such as comorbidities or medications. We used statistical models, such as logistic regression, to calculate individual propensity scores. These scores represented the probability of receiving a treatment or having a specific condition (MB in the current study), determined on the basis of observed covariates. We paired MB patients with non-MB individuals with similar propensity scores [20].

For those in the MB group, the index date was the date of their nearest coronary angiography prior to the establishment of a MB diagnosis; for the non-MB group, it was the date of their earliest coronary angiography within the study period. We followed all patients from their respective index dates until death, the occurrence of study outcomes, the patient’s exit from the insurance system, or the study’s cutoff date (31 December 2018), whichever occurred first.

### 2.3. Categorization of Coronary Artery Disease (CAD)

This study categorized MB and matched non-MB individuals on the basis of their CAD status. Those without ICD codes for CAD were categorized as “no CAD”. Individuals with ICD codes for CAD were subdivided into four groups according to the number of coronary arteries treated. These included insignificant CAD (insigCAD), CAD with single vessel disease (1VD), CAD with double vessel disease (2VD), and CAD with triple vessel disease (3VD). These categories corresponded to percutaneous coronary intervention (PCI) for zero, one, two, and three coronary vessels, respectively. In cases where an individual had undergone multiple PCI procedures, the CAD category was determined by the procedure that treated the most vessels. For example, if an individual first underwent PCI on one coronary artery and then on three, he or she wound be included in the 3VD group. In order to present the real compositions of CAD of MB and non-MB groups, and to conduct sub-analysis in a same-CAD category fashion, the CAD category was not intentionally balanced in terms of selection of study population.

### 2.4. Exposure Definition and Study Outcomes

All diagnoses and study outcomes were identified and defined on the basis of International Classification of Diseases, Ninth Revision, Clinical Modification (ICD-9-CM) and International Classification of Diseases, Tenth Revision, Clinical Modification (ICD-10-CM) codes from 1 January 2016, onward. The diagnosis of MB was established using either a primary or secondary diagnostic code in outpatient or inpatient records (ICD-9-CM code 746.85, ICD-10-CM code Q24.5), of which MB had the highest prevalence [21].

The primary endpoint was a composite of MACEs, including nonfatal myocardial infarction (ST-segment elevation myocardial infarction [STEMI] or non-ST-segment elevation myocardial infarction [NSTEMI]), nonfatal ischemic stroke, and cardiovascular death. Cardiovascular death was defined as a patient having a cardiac-related mortality code or their first three discharge diagnostic codes including STEMI, NSTEMI, ischemic stroke, heart failure, or arrhythmia. The secondary endpoints encompassed a range of conditions, including chest pain requiring a hospital visit, and all-cause mortality. All medical records before and after the index date were reviewed. Patients with a history of nonfatal myocardial infarction, nonfatal stroke, or hemorrhagic stroke prior to a diagnostic code being established for MB, as well as those with unknown sex or survival status, were excluded.

### 2.5. Statistical Analysis

The frequencies and person-years for all study outcomes in the MB and non-MB groups were recorded. Incidence rate ratios and 95% CIs were calculated for each group. The risks of the primary and secondary study outcomes between the groups were estimated using Cox proportional-hazards regression models and propensity scores, and they are presented as HRs with 95% CIs. All statistical analyses were conducted using SAS v.9.4 (SAS Institute, Cary, NC, USA) and R studio. A *p* value of less than 0.05 was considered significant.

## 3. Results

### 3.1. Study Population

**Figure 1:**
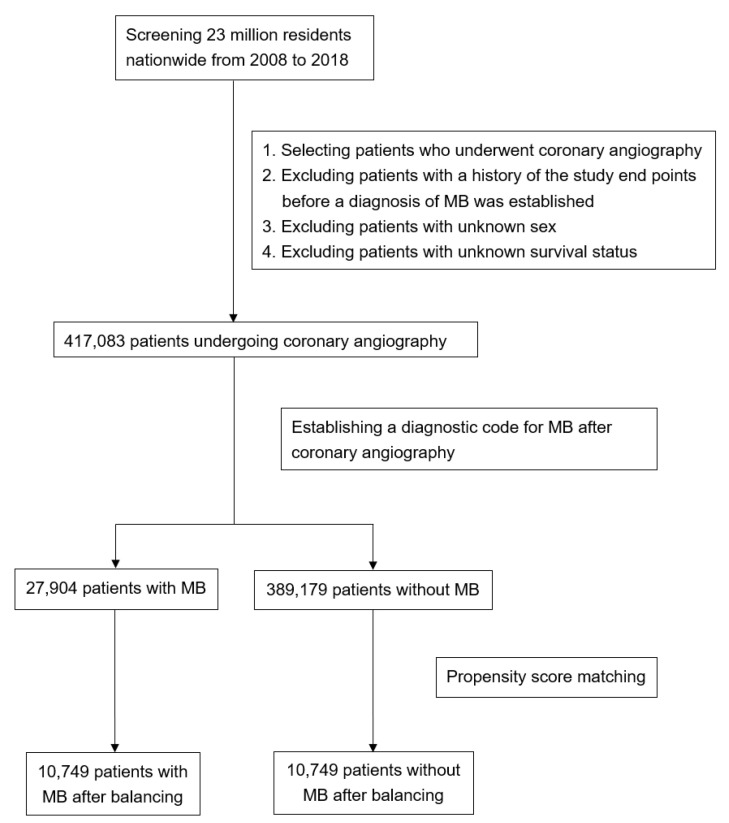
Flowchart depicting selection of cases from the NHIRD for the MB group and that of patients who had undergone coronary angiography.

A total of 27,904 patients with MB and 389,179 patients without MB from January 2008 to December 2018 were identified in the NHIRD. After propensity-score matching was applied to balance the groups, 10,749 patients from each group were included for further analysis (Figure 1). Table 1 presents their baseline characteristics. The mean age was 44.3 years, and the majority of the patients were men (57.3%). No significant differences were observed between the groups in terms of common underlying cardiovascular diseases or medications.

### 3.2. Primary Endpoint and Its Components

**Figure 2:**
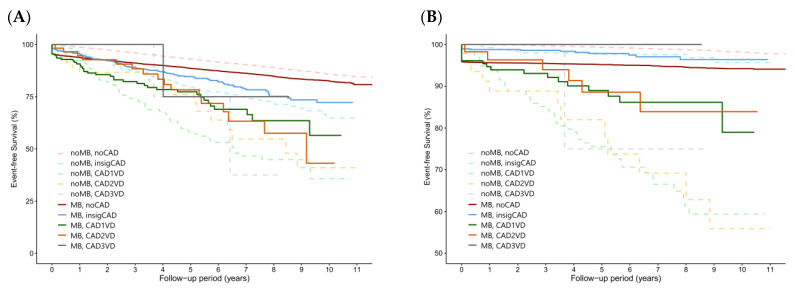

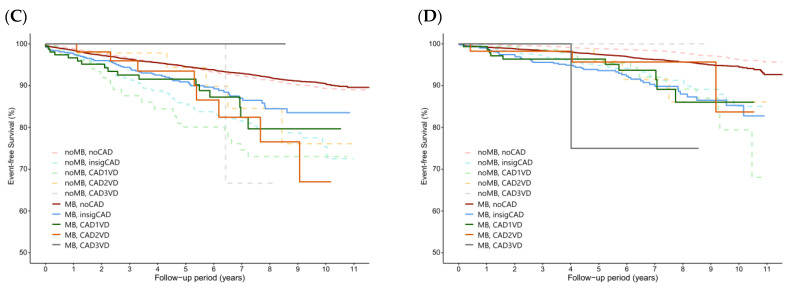
Kaplan–Meier survival curves illustrating the comparison of outcomes between the MB and non-MB groups across different coronary artery disease categories. The analyzed outcomes were (**A**) major adverse cardiovascular events, (**B**) nonfatal myocardial infarction, (**C**) nonfatal ischemic stroke, and (**D**) cardiovascular death.

Over the 11-year study period and mean follow-up period of 5.78 years, primary endpoint events were recorded for 1410 patients (13.12%) in the MB group and 1029 patients (9.57%) in the non-MB group. These patients were further divided into five subgroups according to their CAD category, as illustrated in Figure 2A–D. The *p* values of each pair comparison for Kaplan Meier analysis are provided in Table 2.

**Figure 3:**
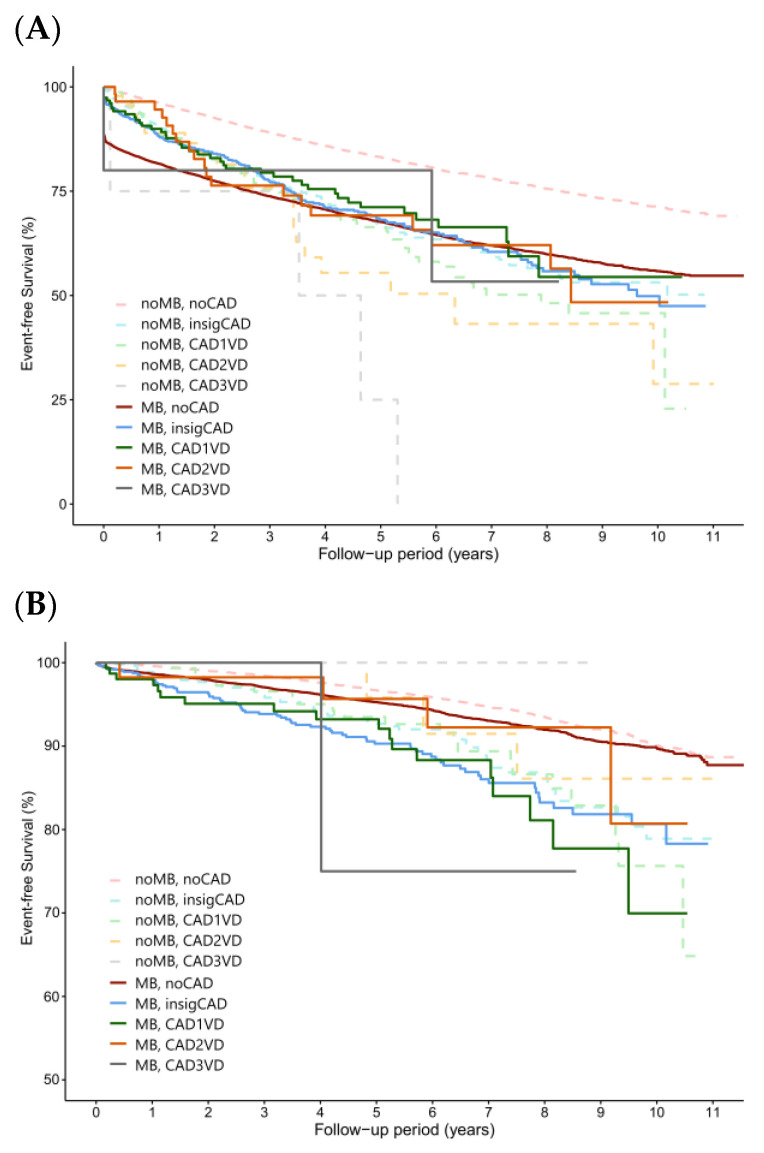
Kaplan–Meier survival curves illustrating the comparison of outcomes between the MB and non-MB groups across different coronary artery disease categories. The analyzed outcomes were (**A**) chest pain necessitating hospital visits and (**B**) all-cause mortality.

The HRs for the study endpoints between different group pairs are detailed in Table 3. In individuals without CAD, MB significantly increased the risk of MACEs, which were primarily driven by increased risks of nonfatal myocardial infarction and cardiovascular death. In addition, the risks of STEMI, NSTEMI, chest pain, and all-cause death increased significantly with MB in these individuals without CAD. However, no significant differences were identified in the risks of nonfatal ischemic stroke, intracranial hemorrhage, and progression to end-stage renal disease necessitating dialysis when the MB (no CAD) and non-MB (no CAD) groups were compared (Table 2 and Figure 3).

No significant differences in the HRs of the study endpoints, with the exception of nonfatal ischemic stroke, were observed between the MB (no CAD) and non-MB groups (insigCAD) (Table 3).

In comparisons with non-MB individuals with 1VD, 2VD, or 3VD, MB (no CAD) did not correlate with elevated risks of MACEs or their components (Table 3). The differences in the MACE risks between the MB (no CAD) and non-MB (1VD through 3VD) groups tended to become greater as the number of atherosclerotic coronary vessels increased in the non-MB groups.

Comparisons of the risks between the MB and non-MB groups with the same CAD categories are presented in Table 2 and Table 4. In the insigCAD, 1VD, 2VD, and 3VD categories, the presence of MB did not significantly increase the risks of MACEs or their components. Additionally, no significant differences were noted in the risks of STEMI, NSTEMI, intracranial hemorrhage, dialysis, and all-cause death between the MB and non-MB groups across the CAD categories (Table 2 and Figure 3).

## 4. Discussion

This is one of the first studies to investigate outcome differences between MB and non-MB groups with varying degrees of CAD. Our findings indicate that in the population without atherosclerotic CAD, MB was associated with an 57% increased risk of a composite endpoint comprising MACEs, particularly nonfatal myocardial infarction and cardiovascular death. Additionally, among the patients without CAD, MB was associated with a higher frequency of chest pain necessitating hospital visits and an elevated rate of all-cause death. The impacts of MB among subjects without CAD from our results were similar to previous research projects in different populations. Yetman et al. [22] reported that MB was associated with a significant increase in symptoms of chest pain and cardiac arrest and with poor survival among children with hypertrophic cardiomyopathy. Sorin et al. [23] revealed that MB was associated with increased risks of MACEs and myocardial ischemia. Kato et al. [18] identified MB as an independent predictor of in-hospital death among patients with Takotsubo cardiomyopathy. In a systematic review and meta-analysis, Bruce et al. [24] reported increased risks of cardiovascular mortality and nonfatal cardiovascular events in MB subjects. In a study focusing on heart transplantation, Tanaka et al. [19] reported an association of MB with accelerated proximal intimal growth and reduced long-term survival.

The current study is among the first to investigate MACEs in patients with MB stratified according to CAD severity. Our results indicate that the effect of MB on clinical outcomes may diminish with the presence and an increase in the severity of CAD. This reduction in MB’s effect on cardiovascular outcomes becomes more pronounced as the severity of CAD increases. For the 2VD and 3VD patients, nearly all study endpoints were statistically identical between the MB and non-MB groups. This indicates that CAD exerts a more substantial influence on cardiovascular outcomes than MB does to the extent that MB does not significantly change clinical events.

Another noteworthy contribution of this study is its comparison of pure MB and different categories of CAD without MB. Our research indicates that the clinical outcomes, with the exception of nonfatal ischemic stroke, of non-MB (insigCAD) are statistically identical to those with pure MB without CAD. Pure MB is essentially equivalent to insignificant CAD. From a treatment perspective, the two conditions require similar therapeutic strategies, including avoidance of coronary stenting, the use of beta-adrenergic antagonists, and the use of antiplatelet agents where indicated. Anatomically and pathologically, MB manifests as a form of nonatherosclerotic CAD. Our results are in line with this perspective.

In the present study, differences in the primary endpoint between the MB and non-MB groups, particularly for individuals in the no CAD category, appeared early and persisted throughout the follow-up period. Atherosclerosis development proximal to the MB segment has been well documented [1,6,25] and can lead to increased risks of cardiac ischemic events after deterioration of coronary patency. This greater atherosclerosis is thought to be promoted by abnormally low shear stress proximal to MB [26,27,28,29].

Studies have provided evidence supporting an association of MB with an increased risk of chest pain, and our findings also support such an association. We observed that MB increased the risk of chest pain only in a normal coronary artery setting; after CAD developed, the presence of MB did not significantly affect clinical symptoms.

The quality of a person’s life would be affected by the frequency of angina. Among subjects without CAD, MB patients presented a significantly higher risk of recurrent chest pain necessitating a hospital visit. Multiple previous observational studies had also noticed this elevated risk of recurrent angina in the MB as compared to the non-MB group [23,30,31]. Nevertheless, when significant CAD developed, the risks of recurrent angina were not significantly different between the MB and non-MB groups. Our result implied that the importance of CAD outweighed that of MB in terms of clinically apparent angina.

The risk of all-cause death in both the MB and non-MB groups paralleled the patterns observed in the aforementioned study endpoints. In the population without CAD, the existence of MB was associated with a 25% higher risk of all-cause death in the MB group relative to that in the non-MB group. However, this increased risk of death in the MB group under the “no CAD” category was not present in the “insignificant CAD” category. The risks of mortality among subjects with significant CAD exceeded that of pure MB significantly (non-MB 1VD and 3VD) and numerically (non-MB 2VD) (Table 3). Our results indicate that the existence of MB does not confer an additional risk on populations with 1VD, 2VD, or 3VD (Table 4). More studies are still needed with the comparative assessment of MB patients’ prognosis related to the degree of severity of bridging.

From this study, we could not explain why MB did not have significant effects in the presence of CAD. We had a hypothesis that the effects of MB were mitigated after the use of medications for CAD, e.g., antiplatelet or lipid-lowering agents. Guidelines for the management of MB are lacking. Antiplatelet or lipid-lowering agents were not absolutely indicated in populations with pure MB in the absence of CAD. Because MB increases shear stress and intimal tear [1], transient endothelial damage and subsequent thrombosis and atherosclerosis may occur. Without antiplatelet or lipid-lowering agents, MB may accelerate thrombotic or atherosclerotic processes. After development of CAD and the use of mandatory medications, the influences of MB may be ameliorated. Prospective, randomized, placebo-controlled trials are needed to guide the optimal management of MB.

MB represents a burden, which cannot be overlooked, to normal physiological circulation in the cardiovascular system. Although MB has traditionally been considered a benign condition, our results demonstrate that it is associated with an increased risk of cardiovascular events in populations without established CAD. Chest pain can have multiple causes, and our results indicate that MB may increase the frequency of chest pain. It remains an unclear question as to how many percentages of chronic coronary syndrome resulted from MB. It needs more detailed surveillance in the future. Because MB is a congenital disease, our results indicate that it is equivalent to insignificant CAD. Individuals with MB can be considered to be born with insignificant coronary artery disease. However, whether early intervention with antiplatelet agents or other atherosclerosis prevention medications would be beneficial for individuals with MB remains unclear.

This study has several limitations. First, our definition of a diagnosis of MB on the basis of *ICD-9-CM* and *ICD-10-CM* codes might have led to underestimation of the prevalence of the condition. Second, this study did not obtain data regarding the precise clinical scenarios and details such as the length, depth, and extent of vascular compression or lumen narrowing in MB segments for each patient. Additionally, the specific coronary vessel (e.g., the right coronary artery, left anterior descending artery, and left circumflex artery) affected by MB in each case was not identified. Patients with 2VD or 3VD were relatively scarce, which restricted the statistical power of the study. In addition, data on smoking status and basic laboratory tests are not available in the NHIRD, which limited our ability to conduct more comprehensive analyses and adjustments.

## 5. Conclusions

Among the population without CAD, the presence of MB significantly increased the risks of major adverse cardiovascular events and all-cause death. However, in the populations with single-, double-, or triple-vessel CAD, the effect of MB gradually diminished as the number of atherosclerotic vessels increased, and clinical outcomes were predominantly influenced by the severity of CAD. From a clinical outcomes’ perspective, MB can be considered equivalent to insignificant coronary artery disease. Further research is required to confirm our findings and to develop novel diagnostic and therapeutic methods for MB.

## Figures and Tables

**Table 1:** Baseline Characteristics of Patients With or Without Myocardial Bridging.

	Before Matching					After Matching				
	No Myocardial Bridging N = 389,179		Myocardial Bridging N = 27,904		SMD	No Myocardial Bridging N = 10,749		Myocardial Bridging N = 10,749		SMD
Age, years	45.37 ± 23.97		52.60 ± 20.48		0.324	44.28 ± 25.30		44.28 ± 25.30		0.000
Sex, male	175,116	45.00	16,508	59.16	0.286	6112	56.86	6206	57.74	0.018
Hypertension, *n* (%)	33,166	8.52	2081	7.46	0.039	854	7.94	952	8.86	0.033
Diabetes mellitus, *n* (%)	79,901	20.53	7387	26.47	0.14	2183	20.31	2061	19.17	0.029
Arrhythmia, *n* (%)	57,298	14.72	9950	35.66	0.497	2304	21.43	2188	20.36	0.026
Hyperlipidemia, *n* (%)	112,907	29.01	13,865	49.69	0.433	4017	37.37	3731	34.71	0.055
Heart failure, *n* (%)	17,893	4.60	3626	12.99	0.3	663	6.17	910	8.47	0.088
Atrial fibrillation, *n* (%)	7289	1.87	1575	5.64	0.199	273	2.54	337	3.14	0.036
CKD, *n* (%)	3400	0.87	388	1.39	0.049	85	0.79	43	0.40	0.051
Cancer, *n* (%)	14,082	3.62	832	2.98	0.036	259	2.41	193	1.80	0.043
CAD, *n* (%)					0.264					0.010
Insignificant	9955	64.23	4466	73.38		571	73.11	748	77.43	
Single vessel	3489	22.51	1134	19.14		159	20.36	154	15.94	
Double vessel	1775	11.45	300	5.06		47	6.02	59	6.11	
Triple vessel	279	1.80	25	0.42		4	0.51	5	0.52	
ACEi or ARB, *n* (%)	78,030	20.05	9991	35.80	0.357	2692	25.04	2585	24.05	0.023
Statin, *n* (%)	78,269	20.11	11,915	42.70	0.502	3183	29.61	3065	28.51	0.024
Aspirin, *n* (%)	99,646	25.60	20,098	72.03	1.049	5648	52.54	5514	51.30	0.025
B-blocker, *n* (%)	54,320	13.96	9734	34.88	0.502	2146	19.96	1777	16.53	0.089
Calcium-channel blocker, *n* (%)	47,166	12.12	7029	25.19	0.34	1659	15.43	1300	12.09	0.097
Thizaide, *n* (%)	6652	1.71	981	3.52	0.114	221	2.06	182	1.69	0.027
SGLT2i, *n* (%)	895	0.23	177	0.63	0.061	35	0.33	36	0.33	0.000
Spironolactone, *n* (%)	16,149	4.15	1852	6.64	0.11	431	4.01	462	4.30	0.015
Metformin, *n* (%)	30,246	7.77	2670	9.57	0.064	830	7.72	809	7.53	0.007
Insulin, *n* (%)	7404	1.90	562	2.01	0.008	168	1.56	145	1.35	0.018
Dabigatran, *n* (%)	712	0.18	146	0.52	0.058	34	0.32	10	0.09	0.051
Apixaban, *n* (%)	222	0.06	77	0.28	0.053	12	0.11	12	0.11	0.000
Rivaroxaban, *n* (%)	1208	0.31	219	0.78	0.064	42	0.39	32	0.30	0.015
Edoxaban, *n* (%)	108	0.03	32	0.11	0.030	6	0.06	7	0.07	0.004
Warfarin, *n* (%)	5689	1.46	942	3.38	0.125	187	1.74	230	2.14	0.029

Note: ACEi, angiotensin converting enzyme inhibitor; ARB, angiotensin receptor blockade; B-blocker, beta adrenergic blocker; CAD, coronary artery disease; CKD, chronic kidney disease; SMD, standardized mean difference; and SGLT2i, sodium glucose cotransporter 2 inhibitor.

**Table 2:** *p* Values for Kaplan Meier Analysis Comparisons.

	Comparison Groups							
	MB (No CAD) versus Non-MB (No CAD)	MB (No CAD) versus Non-MB (1VD)	MB (No CAD) versus Non-MB (2VD)	MB (No CAD) versus Non-MB (3VD)	MB (insigCAD) versus Non-MB (insigCAD)	MB (1VD) versus Non-MB (1VD)	MB (2VD) versus Non-MB (2VD)	MB (3VD) versus Non-MB (3VD)
MACE	<0.0001	<0.0001	<0.0001	<0.0001	0.9999	0.0004	>0.9999	0.9997
Nonfatal MI	<0.0001	<0.0001	<0.0001	<0.0001	>0.9999	<0.0001	0.2187	0.9064
Nonfatal IS	>0.9999	0.0181	0.2841	0.2918	0.6519	0.1043	0.9998	0.9993
STEMI	<0.0001	<0.0001	<0.0001	<0.0001	0.9994	<0.0001	0.0112	0.7382
NSTEMI	<0.0001	<0.0001	<0.0001	<0.0001	>0.9999	0.0083	0.1440	>0.9999
CV death	<0.0001	0.0045	0.0006	0.0003	0.1390	>0.9999	>0.9999	0.7541
Chest pain	<0.0001	<0.0001	<0.0001	<0.0001	>0.9999	0.9917	0.9999	0.9844
All-cause death	<0.0001	0.1955	0.0657	0.0618	0.1550	0.9974	>0.9999	0.9671

Note: CAD, coronary artery disease; CV, cardiovascular; insigCAD, insignificant coronary artery disease; IS, ischemic stroke; MACE, major adverse cardiovascular event; MB, myocardial bridging; non-MB, non-myocardial bridging group; NSTEMI, non-ST-segment elevation myocardial infarction; STEMI, ST-segment elevation myocardial infarction; 1VD, coronary artery disease with single vessel disease; 2VD, coronary artery disease with double vessel disease; and 3VD, coronary artery disease with triple vessel disease.

**Table 3:** Endpoint Hazard Ratios Between MB (Without CAD) and non-MB (With Different CADs).

	Comparison Groups					
	MB (No CAD) versus Non-MB (No CAD)	MB (No CAD) versus Non-MB (insigCAD)	MB (No CAD) versus Non-MB 1VD	MB (No CAD) versus Non-MB 2VD	MB (No CAD) versus Non-MB 3VD	MB (No CAD) versus Non-MB (CAD) 123VD
MACE	1.571 (1.439–1.716)	0.829 (0.638–1.078)	0.250 (0.197–0.318)	0.330 (0.201–0.540)	0.274 (0.068–1.095)	
Nonfatal MI	5.492 (4.401–6.853)	1.011 (0.486–2.102)	0.171 (0.126–0.231)	0.192 (0.108–0.340)	0.210 (0.030–1.497)	0.175 (0.134–0.229)
Nonfatal IS	0.956 (0.855–1.069)	0.679 (0.488–0.946)	0.292 (0.205–0.417)	0.540 (0.224–1.302)	0.305 (0.043–2.164)	0.325 (0.234–0.451)
STEMI	5.493 (4.179–7.220)	0.654 (0.258–1.658)	0.159 (0.111–0.227)	0.170 (0.088–0.329)	0.139 (0.02–0.989)	0.160 (0.116–0.220)
NSTEMI	5.056 (3.670–6.966)	1.845 (0.568–5.993)	0.212 (0.129–0.347)	0.103 (0.054–0.193)	-	0.175 (0.117–0.260)
CV death	1.930 (1.605–2.321)	1.239 (0.810–1.896)	0.366 (0.218–0.615)	0.495 (0.159–1.542)	-	0.398 (0.247–0.639)
Chest pain	2.128 (2.010–2.254)	1.010 (0.826–1.237)	0.877 (0.684–1.123)	0.782 (0.498–1.228)	0.306 (0.115–0.815)	0.830 (0.670–1.026)
All-cause death	1.253 (1.114–1.410)	1.198 (0.842–1.705)	0.522 (0.334–0.815)	0.931 (0.300–2.890)	-	0.590 (0.389–0.895)

Note: CAD, coronary artery disease; CV, cardiovascular; insigCAD, insignificant coronary artery disease; IS, ischemic stroke; MACE, major adverse cardiovascular event; MB, myocardial bridging; non-MB, non-myocardial bridging group; NSTEMI, non-ST-segment elevation myocardial infarction; STEMI, ST-segment elevation myocardial infarction; 1VD, coronary artery disease with single vessel disease; 2VD, coronary artery disease with double vessel disease; and 3VD, coronary artery disease with triple vessel disease.

**Table 4:** Endpoint Hazard Ratios Between MB and non-MB Groups Under Identical CAD Categories.

	Comparison Groups		
	MB (1VD) versus Non-MB (1VD)	MB (2VD) versus Non-MB (2VD)	MB (3VD) versus Non-MB (3VD)
MACE	0.603 (0.408–0.891)	0.796 (0.397–1.597)	0.395 (0.036–4.388)
Nonfatal MI	0.429 (0.246–0.748)	0.385 (0.144–1.026)	-
Nonfatal IS	0.637 (0.353–1.148)	1.374 (0.449–4.204)	-
STEMI	0.442 (0.228–0.857)	0.175 (0.038–0.810)	-
NSTEMI	0.505 (0.209–1.221)	0.339 (0.105–1.092)	-
CV death	1.015 (0.452–2.281)	0.880 (0.177–4.391)	-
Chest pain	0.781 (0.528–1.153)	0.737 (0.387–1.402)	0.169 (0.018–1.562)
All-cause death	1.328 (0.698–2.529)	1.150 (0.256–5.169)	-

CAD, coronary artery disease; CV, cardiovascular; insigCAD, insignificant coronary artery disease; IS, ischemic stroke; MACE, major adverse cardiovascular event; MB, myocardial bridging; non-MB, non-myocardial bridging group; NSTEMI, non-ST-segment elevation myocardial infarction; STEMI, ST-segment elevation myocardial infarction; 1VD, coronary artery disease with single vessel disease; 2VD, coronary artery disease with double vessel disease; and 3VD, coronary artery disease with triple vessel disease.

## Data Availability

Data are not available because of regulations from NHIRD.
